# Mirizzi Syndrome: Clinical Insights, Diagnostic Challenges, and Surgical Outcomes – A 5-Year Experience from a Tertiary Care Hospital in Pakistan

**DOI:** 10.5339/qmj.2025.8

**Published:** 2025-02-23

**Authors:** Hira Moosa, Shabina Jaffer, Muhammad Naeem Khan, Aleena Aftab, Rameez Hussain, Ansharah Mirza, Muhammad Abdul Wasay Zuberi, Anum Iftikhar, Hussain Haider Shah, Saba Patoli, Afnan W.M. Jobran, Farah Hafiz Yusuf, Sameer Abdul Rauf

**Affiliations:** ^1^Department of General Surgery, Jinnah Postgraduate Medical Center Karachi, Pakistan; ^2^Department of Medicine, Dow University of Health Sciences, Karachi, Pakistan; ^3^Department of Medicine, Alquds University, Jerusalem, Palestine; ^4^Department of Oto Rhino Laryngology, Dow University of Health Sciences, Karachi, Pakistan; ^5^Department of Medicine, Liaquat National Medical College, Karachi, Pakistan *Correspondence: Sameer Abdul Rauf. Email: sameerrauf80@gmail.com

**Keywords:** Mirizzi syndrome, laparoscopic procedures, cholelithiasis, laparoscopic cholecystectomy

## Abstract

**Background:**

Mirizzi syndrome (MS) is a rare condition in which the common bile duct or hepatic duct is blocked by impacted gallstones. It can cause symptoms such as cholecystitis, including abdominal pain, nausea, and vomiting. Although diagnosis is challenging, imaging techniques such as ultrasonography and CT scans are helpful. The gold standard for diagnosis is ERCP (Endoscopic Retrograde Cholangiopancreatography). Surgical management is the primary treatment, with laparotomy preferred over laparoscopic procedures.

**Methodology:**

This prospective study was conducted over a period of five years at a tertiary care hospital in Pakistan. A total of 72 patients, aged 21–70 years (mean age 44.81 years), with symptomatic cholelithiasis were included. All patients underwent ultrasonography and, in selected cases, MRCP (Magnetic Resonance Cholangiopancreatography) and ERCP were performed preoperatively. MS was detected preoperatively in 19.4% of cases and intraoperatively in the remaining cases. Data were analyzed using SPSS version 28.

**Results:**

Of the 72 patients, 75% were female. Most patients (69.4%) presented with the right hypochondrium pain, while 16.7% presented with pain and jaundice. Preoperative liver function tests were abnormal in 44.4% of patients. Imaging techniques used included ultrasound (100% of patients), MRCP (22.2%), and ERCP (8.3%). Laparoscopic cholecystectomy was completed in 63.8% of patients, with a conversion rate to open surgery of 30.55%. Two patients required open cholecystectomy with hepaticojejunostomy due to gallstone ileus. The MS types identified were type I (50%), type II (25%), type III (19.4%), type IV (2.77%), and type V (2.77%).

**Conclusion:**

MS is a rare and challenging condition to diagnose. Although imaging techniques are helpful, ERCP remains the gold standard. Surgical management, particularly laparoscopic cholecystectomy, is effective but requires careful implementation by experienced surgeons to avoid complications. In complex cases, laparotomy remains a necessary option.

## INTRODUCTION

Mirizzi syndrome (MS) is a rare outcome of gallstone disease characterized by a blockage of the common hepatic duct (CHD) caused by an impacted gallstone in the cystic duct or gallbladder neck, resulting in extrinsic compression. This condition can cause symptoms such as jaundice, stomach discomfort, and cholangitis. MS is named after Argentine surgeon Pablo Luis Mirizzi, who originally described it in 1948. His research emphasized the need to identify this disease as it can complicate cholecystectomy procedures and has a major impact on the architecture and function of the biliary tract.^1,2^ The incidence ranges from 0.05% to 4%.^3^

Symptoms are similar to cholecystitis and are characterized by dull pain in the right upper abdomen that can spread to the mid-back or the right scapular tip. This pain is usually accompanied by nausea, vomiting, bloating, and gas, with symptoms peaking in the evening. When chronic symptoms persist for a long period of time, there is an increase in the frequency and severity of flare-ups, also known as acute biliary colic. During a medical examination, deep probing of the right upper abdomen may induce pain (known as Murphy's sign). In advanced MS, symptoms and outcomes may be more pronounced.^4^

McSherry et al. classified MS into two types in 1982.^5^ In type I, extrinsic compression of the CHD is caused by a calculus in the cystic duct or Hartmann's pouch, while in type II there is either partial or complete invasion of the calculus into the common bile duct (CBD) resulting in a cholecystocholedochal fistula.^6^ In 1989, Csendes et al. proposed a classification system for MS that further divides patients into four subtypes based on the level of CBD circumference degradation. Type I involves external compression of the CHD, type II involves erosion of less than one-third of the CBD circumference, type III involves erosion of up to two-thirds of the CBD circumference, and type IV involves the destruction of the bile duct.^7^ Beltran et al. introduced type V in 2008 to describe the presence of any of the previous four types as well as the formation of a cholecystoenteric fistula. Type V is further classified into two subtypes: type Va, which does not involve gallstone ileus, and type Vb, which involves gallstone ileus.^8^ However, the existence of multiple classification systems, such as that of McSherry and Csendes, can lead to inconsistency in clinical application. This variation may affect the standardization of diagnostic and treatment approaches, highlighting the need for an accepted, unified classification system in future research.^9^

Due to the inflammatory nature of MS, laboratory tests often show high levels of ALT (Alanine Aminotransferase), AST (Aspartate Aminotransferase), ALP (Alkaline Phosphatase), GGT (Gamma-Glutamyl Transferase), bilirubin, and an increased white blood cell count. Ultrasonography is commonly used to diagnose biliary diseases and can detect gallstones, cholecystitis, and specific symptoms of MS such as an atrophic gallbladder and a dilated CHD with a normal distal CBD, or an edematous gallbladder caused by acute cholecystitis.^10^

Ultrasound, magnetic resonance cholangiopancreatography (MRCP), and endoscopic retrograde cholangiopancreatography (ERCP), in conjunction with a choledochoscope technique, can increase the diagnostic sensitivity of MS. The intraoperative choledochoscope is ideal for identifying MS during surgery.^10^ Computed tomography (CT) scans can help differentiate MS from hepatic tumors or neoplasia, especially in cholecystobiliary fistula.^11^ While imaging modalities such as ultrasound, MRCP, and CT scans are essential for diagnosing MS, they may not always reveal the full extent of the disease, particularly in less pronounced cases. This limitation highlights the importance of intraoperative findings, as MS is often only identified during surgery.^12^

ERCP has long been considered the gold standard for diagnosing MS because it allows for both diagnostic and therapeutic interventions. ERCP has a reported sensitivity rate of 76.2%^13^ and allows direct visualization of the biliary tree via the injection of contrast dye. This technique is especially valuable for identifying impacted gallstones, proximal biliary dilatation, fistulas, and other biliary obstructions.^14^ However, ERCP carries the risk of procedural complications such as pancreatitis, cholangitis, and perforation.

In recent years, endoscopic ultrasound (EUS) has emerged as a valuable adjunct in the diagnosis of MS. EUS provides high-resolution imaging of the biliary system using ultrasound waves transmitted via an endoscope, making it a safer alternative in cases where ERCP may pose higher risks. EUS is particularly useful in assessing biliary strictures, stones, and anatomical variations. Additionally, EUS can be combined with fine-needle aspiration to obtain tissue samples from suspected malignant lesions or masses. MRCP is another non-invasive imaging modality that allows detailed visualization of the biliary and pancreatic ducts without the need for contrast dye injection. When combined with EUS, MRCP has been shown to improve diagnostic accuracy and is increasingly preferred in preoperative planning, reducing the need for invasive procedures such as therapeutic ERCP.^13^ These advances in diagnostic techniques help mitigate risks while ensuring high-quality imaging, particularly in complex cases of MS.^15^

Many patients with MS are not diagnosed until surgery because preoperative diagnosis remains challenging despite advances in imaging techniques. The complex presentation and anatomical variations of the disease can often make diagnosis difficult by imaging alone, with intraoperative findings being crucial for accurate identification.^12^ During surgery, various signs can indicate the presence of MS, including an enlarged or shrunken gallbladder, deformation of Calot's triangle, a lodged gallstone in the cystic duct or gallbladder neck, thick fibrous tissue around Calot's triangle, and adhesions in the subhepatic space. However, surgical management is technically demanding and carries the risk of bile duct injury due to the distorted anatomy frequently observed in this region.^13,16^

Surgical management remains the primary treatment for MS, but is challenging due to the complex anatomy and potential for complications. Recent advances in minimally invasive techniques, such as robotic-assisted laparoscopic cholecystectomy (LC), have improved the precision in navigating the distorted biliary structures associated with MS, helping to reduce conversion rates to open surgery.^17^ However, despite these innovations, laparotomy still offers the advantage of enhanced visualization, but at the cost of a more invasive procedure, higher complication rates, and longer postoperative recovery.^13^ This is partly due to its greater safety compared to the laparoscopic procedure, which has high conversion rates (31–100%) and a higher risk of bile duct damage.^18^ Additionally, it can be particularly difficult for less experienced surgeons to implement detailed surgical strategies, especially when handling advanced cases of MS. The distorted anatomy and risk of bile duct injury require considerable expertise to navigate, highlighting the need for adequate training and experience in these complex procedures.^19^ Total cholecystectomy is recommended for type I MS, while subtotal cholecystectomy or the fundus-first technique is used for type II and type III MS. Type IV MS requires cholecystectomy with Roux-en-Y hepaticojejunostomy, and fistula repair is performed in type V cases. Complications such as bile leaks and retained stones are common problems with surgical management of MS. These issues are particularly relevant when performing bile duct exploration, where inadequate clearance or injury to the duct can lead to significant postoperative morbidity. Surgeons must pay attention to these complications, especially in advanced cases that require more complex biliary reconstruction. The laparoscopic approach is less invasive and offers advantages such as shorter hospital stays and reduced resource utilization. However, when used in MS, conversion rates to open surgery are particularly high. This frequent conversion from laparoscopic to open surgery highlights the limitations of laparoscopic techniques, particularly in complex cases of MS where distorted biliary anatomy and severe inflammation can complicate minimally invasive approaches.^13^ This highlights the need for careful patient selection and readiness for open procedures in advanced stages of MS. In MS, laparoscopic bile duct exploration can be achieved through choledochotomy/transfistula or transcystic approach, although conversion to open surgery is required in 6.8% of cases.^11,13^

The aim of this study was to determine the frequency of MS in cholelithiasis and compare it with developed countries. MS typically occurs in complicated cases of cholelithiasis, often due to delayed surgery and recurrent infections. Furthermore, the study aims to understand the occurrence of these complications in an underdeveloped country and contribute to the knowledge of managing MS. In underdeveloped regions, lack of access to advanced diagnostic modalities such as MRCP and ERCP, along with delays in surgical intervention, often results in more severe cases of MS, such as types IV and V. Limited surgical expertise in managing complex biliary surgeries also contributes to higher complication rates, further complicating the management of MS.^20^ The objective was to examine the frequency, management, and outcomes of MS and to identify appropriate treatment approaches and postoperative complications.

MS poses diagnostic and therapeutic challenges due to the absence of standardized guidelines and consensus on its classification and management. While there are classification systems described by McSherry et al. and Csendes et al., the lack of a universally accepted scheme leads to variability in reporting and treatment approaches.^7,21^ Furthermore, there is limited research comparing the efficacy and outcomes of different surgical techniques for MS. Initially, LC is often attempted, but rates of conversion to open surgery and procedures performed vary depending on the type of MS. The rarity of MS makes large-scale studies difficult and hinders the development of evidence-based guidelines and best practices. Addressing these gaps through comprehensive studies comparing diagnostic accuracy, treatment strategies, and long-term outcomes is essential to optimize patient care and improve understanding of MS management.^22^

## METHODOLOGY

### Ethical approval

The research was conducted in accordance with the Declaration of Helsinki. This study was reviewed and approved by the Institutional Review Board (IRB) of Jinnah Postgraduate Medical Center (JPMC), Karachi (75510) under reference number IRB no. fig2-81/2022-GENL/309/310/JPMC. Permission for data collection was approved by Senior Registrar of JPMC, Karachi. Written informed consent was obtained from the patient for publication and any associated images.

### Study design

A prospective study was conducted at JPMC, Karachi to assess the management and outcomes of MS over a period of five years. The study included 72 consecutive patients who underwent surgery for MS. Patient information, clinical characteristics, preoperative diagnostic methods, surgical procedures, postoperative course, outcomes, and the need for further treatment were documented. Diagnostic criteria were based on preoperative investigations or intraoperative findings using the modified Csendes classification. LC was the initial surgical approach, with conversion to open surgery determined by the surgeon's discretion. In cases with challenging access to Calot's triangle, a fundus-first dissection technique and subtotal cholecystectomy were performed. All patients were followed up for at least six months after surgery, with outpatient clinic visits within the first month.

### Data source

Data for this prospective study were collected by actively documenting information from the medical records of patients admitted to Ward 2 of the Department of General Surgery at JPMC between January 2018 and December 2022. This approach ensured that data were collected in real time when patients were admitted to the ward.

Patients included in this study were approached based on suspicion of having MS, either preoperatively through diagnostic investigations such as ultrasound, MRCP, or ERCP, or intraoperatively when MS was discovered during surgery, which was usually the case. Consent to include their data in the research was obtained postoperatively. Although there were no specific interventions for the study, preoperative ERCP was used when necessary and intraoperative procedures such as T-tube placement or conversion to open surgery were performed as required. The study did not include questionnaires, comparisons, or collection of images.

### Correlating the prevalence of Mirizzi syndrome with risk factors

MS is influenced by several risk factors, including advanced age, obesity, rapid weight loss, fasting, and hormonal variables, particularly estrogen. Gallstone disorder, a common precursor to MS, is more prevalent in the elderly due to prolonged gallbladder stasis and cholesterol oversaturation.^23^ Obesity also leads to the formation of gallstones, which increases the risk of MS because a higher body mass index is related to increased cholesterol release into bile.^24^ Conversely, rapid weight loss or extended fasting can lead to the formation of biliary sludge and gallstones, increasing the risk of MS.^25^ Furthermore, estrogen contributes to the pathophysiology of gallstone disease and makes women more vulnerable to MS, particularly those taking hormone replacement treatment or oral contraceptives.^26^

### Data elements

The study evaluated various surgical techniques used for MS, including LC, laparoscopy converted to open total cholecystectomy, laparoscopy converted to open cholecystectomy with an endo-stapler, laparoscopic converted to open cholecystectomy with T-tube placement, LC with the repair of the cystic area, open cholecystectomy with hepaticojejunostomy, laparoscopy converted to open subtotal cholecystectomy, and laparotomy. We analyzed each reported surgical procedure that resulted in MS and different surgical techniques that influenced the type of MS. The study included patients of all ages with a diagnosis of MS, regardless of gender. Patients with other hepatobiliary disorders who also had MS were excluded to ensure the validity of the results. Patients with gallbladder empyema, gallbladder mucocele, acute pancreatitis, bile duct stricture, and gallbladder polyps were also excluded from the study.

### Data collection procedure

Once approval was granted by JPMC, patients who met the criteria and were admitted to Ward 2 of the Department of Surgery at JPMC, Karachi were included in the study. Data were collected prospectively and a non-probability consecutive sampling technique was used.

### Data analysis procedure

SPSS version 28 was used for data analysis. Frequencies and percentages were calculated for variables such as age and gender and presented in bar and pie charts. Normality was assessed for continuous variables using the Kolmogorov–Smirnov test and the Shapiro–Wilk test. Means were determined for normally distributed variables and medians for non-normally distributed variables. The chi-square test was used to assess the correlation between categorical variables. To assess the correlation between continuous and categorical variables, the t-test and ANOVA were used when the continuous variable was normally distributed. Mann–Whitney U test and Kruskal–Wallis test were used to assess the correlation between continuous and categorical variables if the continuous variable was not normally distributed. The *p* value for significance was considered to be less than 0.05.

## RESULTS

In this study, the participants’ age ranged from 21 to 70 years, with a mean age of 44.81 years and an SD (standard deviation) of 10.71. A total of 1,390 cholecystectomies were performed during the study period, of which 72 (5.17%) patients were diagnosed with MS. Among the patients with MS, the majority were females (*n* = 54, 75%), while males were 18 (25%). Comorbid conditions were observed among the patients, with eight patients (22.8%) having both diabetes mellitus (DM) and systemic arterial hypertension (SAH), 12 (33.4%) patients having only DM, and 14 (38.8%) patients having only SAH. All patients (100%) reported a history of abdominal pain and 16 (22.2%) patients presented with jaundice. Liver function tests (LFT) showed abnormalities in 44.4% of patients, characterized by elevated ALP and GGT levels.

Ultrasound was initially conducted on all patients, which revealed the presence of gallstones in all cases. Choledocholithiasis was detected in two (2.77%) individuals, and ultrasound examination indicated MS in one patient. A CT scan was ordered in one patient to investigate the possibility of a cholecystoenteric fistula, but it failed to confirm the diagnosis. MRCP was performed in all patients with abnormal biliary enzymes and was successful in diagnosing MS in eight (11.11%) cases. ERCP performed in 10 (13.8%) cases involved sphincterotomy for bile duct decompression and placement of bile duct stents. ERCP proved to help diagnose all patients who underwent the procedure. In the remaining patients, MS was diagnosed by direct intraoperative identification of a fistula.

### Types of Mirizzi syndrome

Of the total number of patients, 36 (50%) were classified as type I, 18 (25%) as type II, and 14 (19.4%) as type III. Only two (2.7%) patients were diagnosed with type IV, who underwent an open cholecystectomy and Roux-en-Y hepaticojejunostomy. The remaining two (2.7%) patients were found to have cholecystoenteric fistulas, categorized as type V. Table 1 shows the number of patients with different types of MS.

While this study provides insight into the distribution of different types of MS, the variety of MS types, ranging from type I to type V, complicates interpretation due to the different surgical outcomes and techniques associated with each type. This variation highlights the importance of tailored surgical approaches based on the specific classification of MS, which could contribute to the complexity of comparing outcomes between patient groups.^18^

### Surgical approaches and complications in cholecystectomy for the Mirizzi syndrome

LC was attempted in most patients (94.44%, *n* = 68). However, due to the difficult dissection and challenging anatomy in Calot's triangle, conversion to open surgery was required in 30.55% (*n* = 22) of cases. In a smaller subset of patients (5.6%, *n* = 4), an open technique was selected from the start. Bile duct injuries were managed with selective T-tube repair, rather than as primary treatment for MS, which was performed in eight patients. The overall morbidity rate associated with the procedures was 19.4% (*n* = 7). Bile leaks occurred in five patients, of which three were managed conservatively, while two required ERCP. Additionally, two patients had residual stones that were successfully treated with ERCP and biliary stent placement. These stones were later removed after six weeks with excellent outcomes. One patient experienced duodenal perforation as a complication of ERCP, requiring reoperation. The overall mortality rate was 1.38% (*n* = 1), which was due to chest complications.

The length of hospital stay for the remaining patients varied, and histopathological examination revealed chronic cholecystitis in all cases (100%). Differences in surgical outcomes may have been influenced by several factors, including the surgeon's experience, duration of symptoms before diagnosis, and severity of inflammation or fibrosis around the biliary structures. Additionally, patient-specific factors such as comorbidities and previous abdominal surgeries could have influenced outcomes. A more detailed analysis of these variables is necessary to fully understand their role in postoperative complications and recovery.

All patients were followed up for at least six months, with an average follow-up period of several months. During this period, all patients, except one who underwent hepaticojejunostomy anastomosis, were free of symptoms and had a normal LFT. The exception involved a patient with a persistent increase in serum ALP, who also experienced a mild episode of cholangitis and was successfully treated with antibiotics.

To better understand the factors that influence outcomes in MS, a detailed analysis of patient data was conducted. Patients’ ages ranged from 21 to 70 years (mean 44.81 years), taking gender and comorbidities into account. Of the 72 patients diagnosed with MS, 75% were female and 25% were male. Comorbid conditions such as DM and SAH were present in 60% of the patients, with 38.8% having only SAH, 33.4% having only DM, and 22.8% having both conditions. These comorbidities were associated with higher rates of postoperative complications, particularly bile leaks and longer recovery times, especially in patients with both DM and SAH.

Intraoperative findings such as severe fibrosis and distorted anatomy occurred in patients with advanced MS, contributing to the higher conversion rates to open surgery and increased morbidity. Postoperative recovery data indicated that patients with type I MS had shorter hospital stays than those with more advanced MS types (III and IV), who required more complex procedures such as hepaticojejunostomy and had longer recovery times. This analysis highlights how patient-specific factors, including age, comorbidities, and intraoperative findings, significantly contribute to variability in surgical outcomes.

### Correlation between surgical procedures and the incidence of the Mirizzzi Syndrome

The reported P value of 0.00 indicates a statistically significant correlation between iatrogenic factors and MS. Among the various surgical procedures, laparoscopy converted to open subtotal cholecystectomy and LC were most commonly associated with MS, while laparoscopy converted to open total cholecystectomy with an endo-stapler had the lowest incidence. Further details on the outcomes of other surgical procedures can be found in Table 2.

### Influence of surgical techniques on the types of Mirizzi syndrome

Statistical analysis showed a significant relationship between different surgical techniques and the type of MS (*p* < 0.05). Among the different types, type I MS was predominantly observed as a complication of LC, followed by laparoscopy converted to open subtotal cholecystectomy as the second most common cause. Type II MS showed a relatively equal distribution across all surgical techniques, as shown in Table 3. Type III MS was most commonly associated with open cholecystectomy with hepaticojejunostomy. Only a few cases of type IV MS were found to be associated with LC with the repair of the cystic area, and similarly, a small number of cases of type V MS were identified with laparotomy and laparoscopy converted to open subtotal cholecystectomy. Table 3 shows the impact of different surgical techniques on the types of MS.

## DISCUSSION

MS occurs rarely in only 0.7–5% of patients undergoing cholecystectomy and in 0.1% of patients with cholelithiasis. In developing countries such as Asia and Central and South America, it occurs in 4.7–5.7% of patients.^27–29^ In the present study, the incidence of MS was 5.17%.^19,30^. The reason may be delayed surgery due to long waiting list for cholecystectomies and ignorance of complications of cholelithiasis due to poverty. However, these interpretations may vary depending on factors such as the availability of healthcare resources, diagnostic capabilities, and expertise of surgical teams in different regions. The variability in access to early diagnosis and treatment could influence the incidence and severity of MS, and caution should be exercised when generalizing these results to other settings.

This syndrome usually has female predominance, with this series supporting this majority. We had a mean age of 44.81 years, according to the literature.^31^. Accurate preoperative diagnosis is the main challenge in the management of MS, which is achieved in 8–62% of cases. If this is not achieved, the rate of bile duct damage could be as high as 17%.^6^ As our radiologists are still in the learning curve to diagnose MS, it is easily confused with cholangitis, choledocholithiasis, and sometimes with gall bladder malignancy. We were able to make a preoperative diagnosis in 27.77% of patients: two via ultrasound, eight with MRCP, and 10 with ERCP.

Although ERCP has 100% sensitivity, it is invasive and can cause side effects such as hemorrhage, pancreatitis, duodenal perforation, sepsis, and failure to cannulate CBD in 6–22% of cases.^2,13,32^ Owing to the lack of ERCP at our institution, it was performed outside of our hospital. It was difficult to make the preoperative diagnosis. In patients with a high suspicion of MS, non-invasive MRCP was ordered, which showed a narrow CBD in two patients and the impression of MS in six patients.^33^

Although imaging studies are considered to be the most sensitive and specific if MS is suspected, more than 50% of diagnoses are made intraoperatively. In our study, the diagnosis was made intraoperatively in 72.23% of patients, resulting in bile duct injury in 27.77% of patients, most of which were grade 1 injuries, and CBD was repaired over a T-tube in 11.11% of patients. Although interest in this topic has increased due to advances in biliary tract surgery and an increase in the prevalence of cholelithiasis, more consensus on the diagnosis or surgical treatment strategies for MS is still needed.^34,35^ This depends mainly on the experience and ability of the operating surgeon to modify their dissection strategy and use available instruments and techniques. Once the diagnostic criteria of MS are identified and the diagnosis is made, the surgeon must make decisions that will facilitate cholecystectomy, deal with difficult bile duct stones, and minimize the risk of potentially serious complications, particularly bile duct injury. Several factors not fully explored in this study may also influence the management and outcomes of MS, including the surgeon's experience, the availability of advanced diagnostic tools such as intraoperative cholangiography, and the patient's overall health status. These factors can affect surgical decision-making, postoperative recovery, and the risk of complications, suggesting that further investigation is needed into their specific role in optimizing MS management.^18^

We performed LC in 46 (63.88%) patients, with an endo-stapler in six patients and with the repair of a cystic area in eight patients, including 34 (47.21%) patients of MS type I, eight (11.11%) patients of MS type II, and four (5.55%) patients of MS type III. Conversion occurred in 22 (30.55%) patients, with T-tube placement in eight (11.11%) type II and III patients, laparoscopic converted to open with subtotal cholecystectomy in 10 (13.88%) type II and III patients, and laparoscopic converted to open total cholecystectomy in four (5.55%) type I and II patients. Open cholecystectomy with hepaticojejunostomy was performed in both patients with type IV MS and laparotomy was performed in both patients with gallstone ileus.

Our literature search found that surgery is the recommended treatment for MS, and Paul et al. were the first to complete LC for type I MS.^36,37^ There are surgical approaches to deal with MS, either the open or laparoscopic approach, and there is debate about which approach is better.

According to the systemic reviews by Antoniou and Hang Chen, although open surgery is more invasive, has a high complication rate, and has a longer postoperative hospital stay, most surgeons prefer it as a treatment method for MS because it provides better visualization, tactile input, and removal of gallbladder calculus before cholecystectomy. The laparoscopic method is not preferred due to an increased risk of bile duct injury and a high conversion rate (11–100%). Furthermore, the laparoscopic approach should be limited to type I and should only be performed by an experienced biliary surgeon.^13,38^. Some authors indicate that LC is contraindicated in MS and that the recommended treatment options for an open surgical approach include a total cholecystectomy for type I, leaving the portion of the infundibulum adherent to the CBD, a retrograde fundus-first cholecystectomy or a partial (subtotal) cholecystectomy for types II and III, and a portion of the infundibular wall is used for CBD closure when significant inflammation prevents the safe dissection of Calot's triangle.^32,35^

A Roux-en-Y hepaticojejunostomy is recommended for type IV and a laparotomy for type V. When the integrity of the tissue repair is in doubt, inserting a T-tube into the bile duct will decompress it and reduce the risk of bile leakage.^39–41^ The T-tube can also be used to remove the remaining stones by interventional radiology, but this is less good compared to leaving a portion of the gallbladder infundibulum or cystic duct that can be used to repair the CBD fistula (choledochoplasty), a technique applicable in MS type II and selected type III cases.^42–44^ However, choledochal-enteric anastomosis is a preferred alternative to this method because strictures may occur that require endoscopic treatment.^45,46^

In our study, most of the patients had MS type I and we were able to perform the procedure laparoscopically. If we encountered difficulties, we either performed a subtotal cholecystectomy or used an endo-stapler to complete the procedure laparoscopically. The conversion rate was 30.55%, and we completed the surgery laparoscopically in 63.88% of cases. For MS type IV, we chose an open approach and performed a Roux-en-Y hepaticojejunostomy in both cases. For type V, we performed an open midline laparotomy in both patients, as recommended in the literature. Initially, we tried a laparoscopic approach for all MS types (I, II, and III) and completed the surgery laparoscopically in 63.88% of cases. However, in some cases, we had to switch to an open approach. Only 22 patients required conversion to an open approach, with total cholecystectomy performed in two type I cases. For type II, we performed laparoscopic converted to open with total cholecystectomy in two patients, laparoscopic converted to open subtotal cholecystectomy in four patients, and laparoscopic converted to open cholecystectomy with T-tube placement in four patients. For type III, laparoscopic converted to open subtotal cholecystectomy was performed in six patients, and laparoscopic converted to open cholecystectomy with a T-tube was performed in four patients.

There are only a few papers that describe experiences with the laparoscopic technique for the treatment of MS. The majority of these were case studies or case reports. Kumar et al. preferred open surgery because they were only able to successfully complete the procedure laparoscopically in one out of 23 patients.^47^

Despite the risks and associated technical difficulties, some experts continue to recommend LC and laparoscopic subtotal cholecystectomy (LSC) because the surgeon loses laparoscopic view in open surgery. Kamalesh et al.^48^ were successful in performing LC in 70% of patients and bilioenteric anastomosis with T-tube placement for patients with types II and III.^47,49^ They recommended a subtotal cholecystectomy. Rohatgi and Singh^49^ had success with LSC in MS, but recommended that intraoperative cholangiography is mandatory for such cases. Yetişir et al. successfully performed LSC along with resection of a cholecystocolic fistula and application of Tri-staples for MS type V.^16,50,51^

In the present study, the laparoscopic approach was successful in 63.88%, which was comparable to the study by Kamalesh, where we adopted an endo-stapler and subtotal cholecystectomy due to the difficulty in performing total cholecystectomy and conversion, and the T-tube was used in types II and III, which was again comparable to the existing literature. Intraoperative cholangiogram was not available in our hospital.

## LIMITATIONS

To ensure accurate analysis, we excluded some patients who were lost to follow-up from the study. We compared our results only with the existing literature and did not use other methods such as laparoscopic repair or hepaticojejunostomy. Due to the rarity of the disease, obtaining an appropriate sample size was challenging, which may have limited our ability to identify clear patterns in the presentation and management of the syndrome from the available data. It is important to note that our study only represents specific disease characteristics and may be subject to bias. There are no clear guidelines for the management of MS, so surgical and healthcare practices may vary. As a cross-sectional study, we did not compare our management practices to a control group, and therefore the effectiveness of our strategies cannot be definitively assessed. We did not exclude patients with comorbidities due to the rarity of the syndrome, which may have affected management outcomes.

## CONCLUSION

In MS type I, II, or III cases, LC is often the preferred approach, with the option of converting to open surgery if needed. However, for MS type IV, an open approach with hepaticojejunostomy is often considered the most suitable choice. In cases where acute obstruction is present, particularly in MS type V, laparotomy is typically recommended to address the condition effectively. Understanding the specific type of MS is essential for tailoring the surgical approach to ensure optimal patient outcomes.

### List of abbreviations

[Table tbl4]

### Competing interests

The authors have no conflicts of interest to declare.

### Authors’ contribution

**HM**, **SJ**, and **MNK**: Contributed to conceptualization, wrote the original draft, gave final approval, and ensured the accuracy of the work. **AA**, **RH**, **AM**, **MAWZ**, **AI**, **HHS**, and **SP**: Wrote the original draft, gave final approval, and ensured the accuracy of the work. **AWMJ**, **FHY**, and **SAR**: Involved in reviewing and editing. All authors read and approved the final version of the manuscript.

## Figures and Tables

**Table 1.** Number of patients with different types of Mirizzi syndrome (MS).

Type of MS	No. of patients (*n*)	%

Type I	36	50

Type II	18	25

Type III	14	19.4

Type IV	2	2.77

Type V	2	2.77


**Table 2.** Correlation between surgical procedure and Mirizzi syndrome (MS).

Surgical technique	Intraoperative conversion to MS		

	Yes (*n*/%)	No (*n*/%)	Total (*n*/%)

LC	0/0	32/100	32/100

Laparoscopy converted to open total cholecystectomy	4/100	0/0	4/100

Laparoscopy converted to open cholecystectomy with an endo-stapler	0/0	6/100	6/100

Laparoscopic converted to open cholecystectomy with T-tube placement	7/87.5	1/12.5	8/100

LC with the repair of the cystic area	2/25	6/75	8/100

Open cholecystectomy with hepaticojejunostomy	2/100	0/0	2/100

Laparoscopy converted to open subtotal cholecystectomy	9/90	1/10	10/100

Laparotomy	0/0	2/100	2/100


**Table 3.** Impact of surgical techniques on the types of Mirizzi syndrome (MS).

Surgical technique	Type of MS					

	Type I (*n*/%)	Type II (*n*/%)	Type III (*n*/%)	Type IV (*n*/%)	Type V (*n*/%)	Total (*n*/%)

LC	30/93.8	2/6.3	0/0	0/0	0/0	32/100

Laparoscopy converted to open total cholecystectomy	2/50	2/50	0/0	0/0	0/0	4/100

Laparoscopy converted to open cholecystectomy with an endo-stapler	0/0	2/33.3	4/66.7	0/0	0/0	6/100

Laparoscopic converted to open cholecystectomy with T-tube placement	0/0	4/4	4/4	0/0	0/0	8/100

LC with the repair of the cystic area	4/50	4/50	0/0	0/0/	0/0	8/100

Open cholecystectomy with hepaticojejunostomy	0/0	0/40	0/60	2/0	0/0	2/100

Laparoscopy converted to open subtotal cholecystectomy	0/0	4/40	6/60	0/0	0/0	10/100

Laparotomy	0/0	0/0	0/0	0/0	2/100	2/100


**Table tbl4:** 

CBD	Common Bile Duct

DM	Diabetes Mellitus

ERCP	Endoscopic Retrograde Cholangiopancreatography

LC	Laparoscopic Cholecystectomy

LFT	Liver Function Tests

LSC	Laparoscopic Subtotal Cholecystectomy

MRCP	Magnetic Resonance Cholangiopancreatography

MS	Mirizzi Syndrome

SAH	Systemic Arterial Hypertension

SD	Standard Deviation

